# Environmental controls on butterfly occurrence and species richness in Israel: The importance of temperature over rainfall

**DOI:** 10.1002/ece3.7969

**Published:** 2021-08-02

**Authors:** Orr Comay, Oz Ben Yehuda, Racheli Schwartz‐Tzachor, Dubi Benyamini, Israel Pe'er, Inbar Ktalav, Guy Pe'er

**Affiliations:** ^1^ Department of Ecosystem Services UFZ Helmholtz Centre for Environmental Research Leipzig Germany; ^2^ German Centre for Integrative Biodiversity Research (iDiv) Halle‐Jena‐Leipzig Leipzig Germany; ^3^ School of Zoology and the Steinhardt Museum of Natural History Tel Aviv University Tel Aviv Israel; ^4^ Achva Academic College Arugot Israel; ^5^ Ramat Hanadiv Zikhron Ya'akov Israel; ^6^ Israeli Lepidopterists Society Bet Arye Israel; ^7^ GlueCAD‐Biodiversity IT BMS‐IL Web‐portal Haifa Israel; ^8^ Department of Archaeology Laboratory of Archaeozoology University of Haifa Haifa Israel

**Keywords:** biogeography, bioindicators, butterflies, citizen science, community ecology, generalized linear latent variable model

## Abstract

Butterflies are considered important indicators representing the state of biodiversity and key ecosystem functions, but their use as bioindicators requires a better understanding of how their observed response is linked to environmental factors. Moreover, better understanding how butterfly faunas vary with climate and land cover may be useful to estimate the potential impacts of various drivers, including climate change, botanical succession, grazing, and afforestation. It is particularly important to establish which species of butterflies are sensitive to each environmental driver.

The study took place in Israel, including the West Bank and Golan Heights.

To develop a robust and systematic approach for identifying how butterfly faunas vary with the environment, we analyzed the occurrence of 73 species and the abundance of 24 species from Israeli Butterfly Monitoring Scheme (BMS‐IL) data. We used regional generalized additive models to quantify butterfly abundance, and generalized linear latent variable models and generalized linear models to quantify the impact of temperature, rainfall, soil type, and habitat on individual species and on the species community.

Species richness was higher for cooler transects, and also for hilly and mountainous transects in the Mediterranean region (rendzina and Terra rossa soils) compared with the coastal plain (Hamra soil) and semiarid northern Jordan Vale (loessial sierozem soil). Species occurrence was better explained by temperature (negative correlation) than precipitation, while for abundance the opposite pattern was found. Soil type and habitat were insignificant drivers of occurrence and abundance.

Butterfly faunas responded very strongly to temperature, even when accounting for other environmental factors. We expect that some butterfly species will disappear from marginal sites with global warming, and a large proportion will become rarer as the region becomes increasingly arid.

## Graphical abstract

While butterflies are often used as bioindicators for the effects of climate and land‐cover change on biodiversity, their response to these factors was little studied outside temperate habitats. Here, we use Citizen Science data to demonstrate that temperature is the lead driver of butterfly biodiversity in Israel, followed by rainfall. Land cover and soil type had lesser, although significant, impacts on butterfly biodiversity.

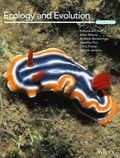

## 1 INTRODUCTION

Rapid climate and land‐use changes drive strong responses of species (abundance, distributions), communities (richness, species composition), and ecosystems (functions, services). Owing to their exceptional diversity and contribution to key ecosystem functions and services (IPBES, 2016), recent studies have focused on the decline of insects (Hallmann et al., 2017; Rada et al., 2019 Van Klink et al., 2020). Long‐term systematic monitoring is critical for assessing trends and informing policymakers (Didham et al., 2020).

Assessing trends in a rich and varied group such as insects can be facilitated by focusing on particular taxa as bioindicators. Criteria to define good bioindicators are a tractable taxonomy, sensitivity to environmental changes, rapid and observable response (hopefully faster than other groups), sufficient ecological knowledge to allow inferring from responses to causes, and a potential to inform also on the status and trends of other groups that are not monitored (Syaripuddin et al., 2015).

Butterflies are considered as important climate and habitat bioindicators (Parmesan, 2003; Pe'er & Settele, 2008 and references therein). They are diverse, easily detectable, their biology and ecology were well studied, and they have been recorded intensively enough by experts and volunteers to establish a strong knowledge‐base to start with. Accordingly, they have been among the first taxa to demonstrate phonological and distributed changes in response to climate, which was also found to be consistent with other taxonomic groups (Parmesan et al., 1999 and references therein, Parmesan, 2006). More recently, Høye et al. (2014) found that butterfly flight season advances with earlier snowmelt and higher temperatures in Greenland. Butterfly faunas changed faster than birds with rising temperatures in Europe (Devictor et al., 2012), and Herrando et al. (2016) found that butterflies abundance responded faster than birds to land abandonment in Iberia. Comparing four taxonomic groups in Malaysia, Syaripuddin et al. (2015) found butterflies to be the most promising group as bioindicators. Inference potential was demonstrated for example by Roy et al. (2001): When training models correlating weather and butterfly abundance in the 20th century, they could successfully reproduce patterns of change in butterfly abundance back to the 19th century. Oostermeijer and van Swaay (1998) demonstrated also that butterfly community also responds to soil conditions. It is likely due to their short life cycles that they demonstrate rapid response to environmental change, whereas their variable levels of dependency on specific host plants leads to relatively strong response to local environmental conditions. More specifically, some species feed as larvae on a wide range of host plants (e.g., *Vanessa cardui*), while others are highly specific to a single plant, habitat, and/or ant species. Their climatic response likely emerges from the plasticity of some species in terms of flight period phenology (e.g., *Anthocharis cardamines* flies in May–July in Britain (Courtney & Duggan, 1983), compared with February–March in Israel (Benyamini, 2010)).

However, there are limitations to the use of any single taxonomic group as bioindicators, and these must be assessed and critically evaluated—especially for insects given their stochastic population dynamics (Gerlach et al., 2013). For instance, Pellissier et al. (2020) found stronger signals of abundance response of birds to Natura‐2000 cover in Europe, compared with butterflies (albeit, potentially resulting from both difference in habitat affiliation and scale of response); and Maleque et al. (2009) proposed that different taxa should be used complementarily, to address different contexts and responses.

Efficient use of butterflies as bioindicators, in the right contexts and manner, requires deeper understanding of the environmental drivers of their occurrence and abundance, at both the species and community levels (Fleishman & Murphy, 2009); and evaluating the strengths, weaknesses, limitations and potential realms of applicability in different contexts. This was not frequently done. Several studies have considered biogeographical patterns in butterfly faunas on regional scales and studied the impacts of climate and latitude; however, far fewer studies have examined the impacts of local conditions (e.g., land cover; Dapporto et al., 2017; Hawkins, 2010; Stefanescu et al., 2004). Some studies have focused on local impacts in addition to climate, but only on the local scale (Checa et al., 2014; Gutiérrez Illán et al., 2010; Horner‐Devine et al., 2003). Others have studied the impacts of both climate and habitat on butterfly occurrence and species richness, but not on abundance and community composition (Kivinen et al., 2007; Newbold et al., 2009). Furthermore, beyond the initial indication that soils may shape butterfly faunas (Oostermeijer & van Swaay, 1998), this area remains understudied. Finally, to the best of our knowledge, no study has explored the impacts of both climatic gradients and local environment (habitat and soil) on butterflies on a national scale.

To advance the knowledge regarding the environmental factors affecting butterflies, in this study, we aimed to characterize Israel's butterfly fauna (species and communities) in terms of biogeography, occurrence, abundance, and habitat affiliation. We identified the main environmental drivers behind observed patterns, using data collected by participants of the Israeli Butterfly Monitoring Scheme (BMS‐IL). This study was motivated by Israel's high climatic heterogeneity and absence of systematic butterfly habitat affiliation analysis. We particularly wanted to assess whether species occurrence and abundance are driven more by climate (temperature, rainfall) or by land use (habitat, soil type). Therefore, we considered to what extent butterflies can be used as climate or habitat indicators.

## 2 METHODS

### 2.1 Study area

**FIGURE 1:**
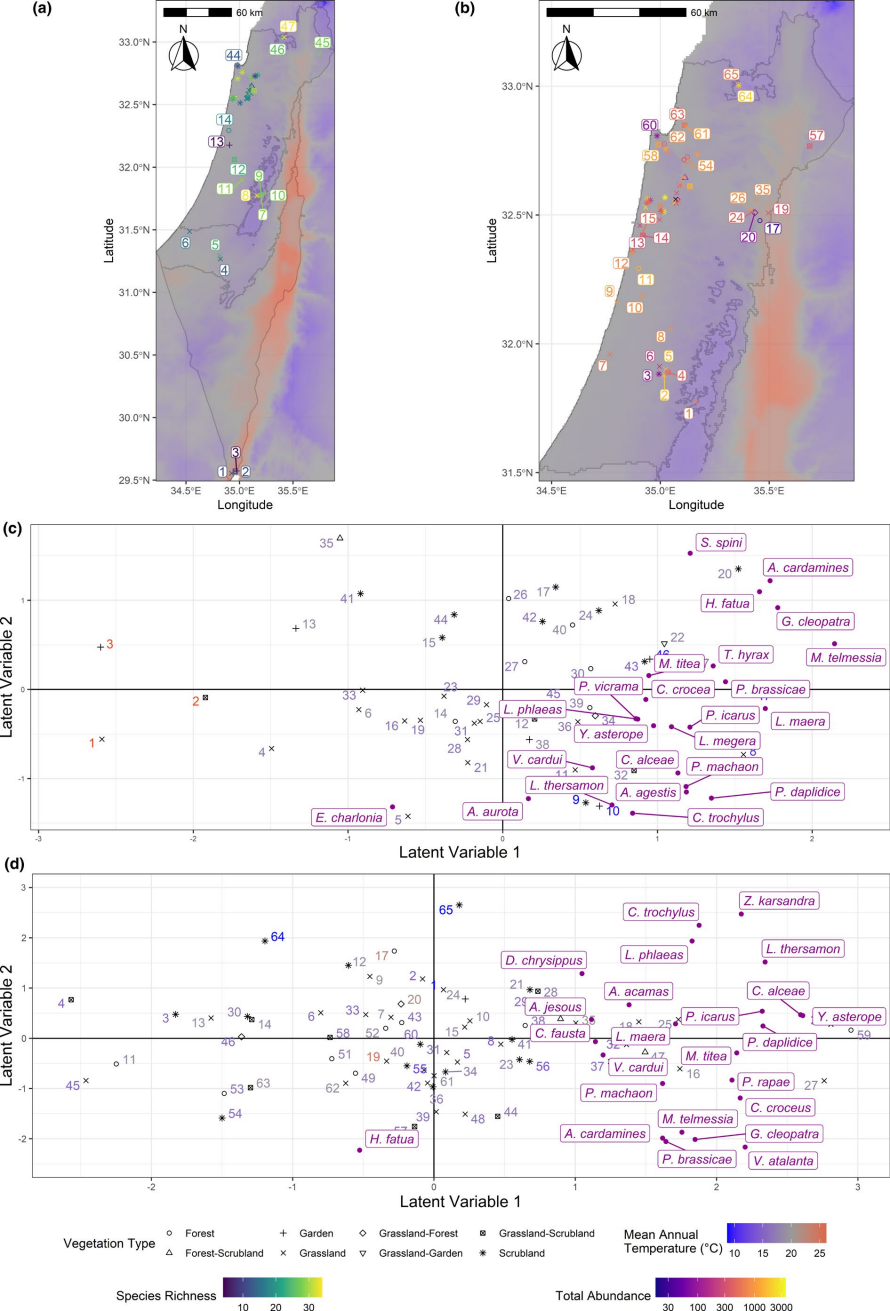
Maps of Israeli butterfly species richness (a) and total butterfly abundance (b) per transect, and ordinations of transects and species by occurrence (c) and abundance (d). Transects are numbered from south to north, once for occurrence and once for abundance. Ordination axes are latent variables, and similarity in species occurrence/abundance decreases with distance (e.g., transects with more similar species are drawn closer together). Species tending to co‐occur (or reaching relatively high abundances) in the same transects are drawn closer together. Shading denotes (a) species richness, (b) total butterfly abundance, and (c and d) mean annual temperature. Background color in (a) and (b) is the mean annual temperature. Point shape depicts habitat (vegetation type)

The study area covered Israel, including the West Bank and Golan Heights (Figure 1a). The climate is largely Mediterranean, with most precipitation occurring in winter; there is a sharp north–south aridity gradient (Israel Meteorological Service, 2020), with ~900 mm/annum in the northern mountains and <30 mm/annum in the southern Wadi Araba. We thus divided the study area into four ecoregions: desert (<200 mm rain/yr), semidesert (200–350 mm), low‐lying Mediterranean (>350 mm rainfall/yr, <700 m above sea level [a.s.l.]), and high Mediterranean (>350 mm rainfall/yr, 700–1,300 m a.s.l.). Mt. Hermon (1,300–2,224 m a.s.l) was excluded as it contains no BMS‐IL transects. For a map of the study area, climatic conditions, and transect distribution, see Appendix S1, Figure S1.1 and Table S1.

### 2.2 Soil types

Soil types in the study area are detailed in Singer (2007) and are summarized in Table S1.2 Appendix S1. Soil maps are only available as ‘soil associations’, which include several intermingled soil types (Table 9.4‐1 in Singer, 2007). Note that while soil types are dependent on climate, they are also heavily dependent on other factors—particularly geology and topography, as well as vegetation (Singer, 2007).

### 2.3 Environmental data

We extracted mean annual temperature and mean annual rainfall for each transect from Geographic Information System (GIS) raster layers with a cell edge size of 30 arcseconds (~924 by ~796 m in the study area), downloaded from WorldClim version 2 (Fick & Hijmans, 2017). We extracted soil type per transect from a polygonal GIS layer of soils obtained from the Israeli Ministry of Agriculture and Rural Development, which we converted to a raster layer with a cell edge size of 30 arcseconds. We assigned each transect the soil type of the start of its first section.

We used the main categories of the European Nature Information System (EUNIS, 2020) to classify habitats: (E) grasslands and lands dominated by forbs, mosses, or lichens (hereafter ‘grassland’); (F) heathlands, scrub, and tundra (hereafter ‘scrubland’); (G) woodland, forest, and other wooded land (hereafter ‘forest’); and (I) regularly or recently cultivated agricultural, horticultural, and domestic habitats (hereafter ‘garden’, as no transects were located in agricultural habitats).

For each 50‐m section, we defined the habitat category based on satellite images and maps. Where possible, we also used ground‐truthing through personal familiarity with the sites, a visual inspection of aerial photographs, and through communication with transect volunteers where need be. We then defined the overall habitat of each transect according to the frequency of habitat classes in its sections. In cases where at least 75% of the sections fell under one habitat class, the entire transect was attributed to that class. Otherwise, transects were regarded as having mixed habitats, based on the two dominant classes. No transect required an assignment of more than two habitat classes (i.e., together covering less than 67%).

### 2.4 Butterfly data

The dataset used for our analyses originates from the Israeli Butterfly Monitoring Scheme (BMS‐IL). Founded by the Israeli Lepidopterist Society in 2009, BMS‐IL follows the same principles as other schemes, focusing on Pollard‐walk transects (Pollard, 1977; Taron & Ries, 2015; van Swaay et al., 2015). Transects in BMS‐IL are 300–600 m long, subdivided into 50‐m sections. While they usually cover a single habitat type, some heterogeneity is unavoidable. Volunteers visit transects in dry weather with gentle or no winds, and when temperatures are above 17℃ (or above 13℃ with direct sunshine). Observers register the number of adult individuals of all species observed (sometimes only genus/family) in an imaginary 5 × 5 × 5 m cube (van Swaay et al., 2015). If no butterflies are observed in a given visit, observers report ‘none seen’. BMS‐IL requests that volunteers perform observations twice per month year‐round, with an optional break in July–September (see Schmucki et al., 2016 for justification of visitation frequency). For a detailed description of BMS‐IL, see Comay et al. (2020).

### 2.5 Analysis of species occurrence and richness

Volunteers joined BMS‐IL at different times, and hence, some have recorded more species than others. To rectify for potential biases due to such differences in sampling efforts, we first examined 14 transects that were monitored for at least 10 years. We compared the proportion of species accumulated every year with the final species richness after 10 years; from this, we identified the number of monitoring years needed to obtain a representative species list per transect. After 5 years of monitoring, 75% of the transects recorded more than 80% of the species that were eventually recorded in 10 years, half recorded more than 90% of the final species richness, and none had less than 75% of their final species richness (Appendix S1, Figure S1.2). Moreover, inter‐transect variation in the percentage of species observed in each transect, compared with the total species list after 10 years of monitoring, diminished quickly with time, likely due to increasing volunteer experience. Thus, to analyze species' occurrence, we generated species lists using the first 5 years of data from all transects that allowed it. Transects with <5 years of monitoring were excluded. This procedure generated a list of 73 species from 47 transects. To avoid overfitting models to very rare species, we included only species that occurred along at least four transects. This procedure generated a list of 49 species for statistical analysis (Table 1). To assess species' occurrence and richness per ecoregion (without statistical testing), we included all 73 species.

**TABLE 1:** Israeli butterfly species analyzed for occurrence and abundance

Species^a^		Occur. transects^b^	Abund.^c^ (mean % ± *SD* where it occurs)	No. of occurrence transects^d^				Europe (Wiemers et al., 2018)
				Wadi Araba	Semi‐desert	Low Med.	High Med.	
All transects		47	65	3	3	36	5	NA
Total species		73	24	18 (25%)	32 (44%)	56 (77%)	57 (78%)	58 (79%)
Unique species		NA	NA	2	1	6	11	NA
Mean annual rainfall (mm)		NA	NA	15	277	585	589	NA
Mean annual temperature (°C)		NA	NA	22.8	19.2	19.0	16.6	NA
Family: Papilionidae (4 species)				0 (0%)	2 (50%)	3 (75%)	3 (75%)	3 (75%)
*Papilio machaon*	39		716 (3% ± 3%)	–	3	31	5	Occurs
*Allancastria cerisyi*	7		Not analyzed	–	–	7	–	Occurs
*Allancastria deyrollei*	1		Not analyzed	–	–	–	1	–
*Archon apollinus*	27		Not analyzed	–	1	25	1	Occurs
Family: Pieridae (16 species)				7 (47%)	9 (60%)	13 (87%)	13 (87%)	12 (75%)
*Aporia crataegi*		2	Not analyzed	–	–	1	1	Occurs
*Pieris brassicae*		41	2,698 (12% ± 12%)	–	2	34	5	Occurs
*Pieris rapae*		42	2,365 (11% ± 14)	1	3	33	5	Occurs
*Pontia daplidice*		39	2,130 (5% ± 6%)	–	3	31	5	Occurs
*Pontia glauconome*		5	Not analyzed	3	–	2	–	–
*Colotis fausta*		44	3,402 (18% ± 24%)	2	1	36	5	–
*Colotis phisadia*		3	Not analyzed	3	–	–	–	–
*Anaphaeis aurota*		33	Not analyzed	2	2	27	3	–
*Euchloe ausonia*		19	Not analyzed	–	1	16	1	Occurs
*Euchloe belemia*		28	Not analyzed	–	3	22	3	Occurs
*Anthocharis cardamines*		30	275 (2% ± 5%)	–	–	26	4	Occurs
*Anthocharis damone*		1	Not analyzed	–	–	–	1	Occurs
*Colias crocea*		41	537 (2% ± 2%)	–	3	33	5	Occurs
*Gonepteryx cleopatra*		30	495 (3% ± 3%)	–	–	25	5	Occurs
*Euchloe charlonia*		4	Not analyzed	1	2	–	1	Occurs
*Catopsilia florella*		10	Not analyzed	2	–	8	–	Occurs
Family: Nymphalidae (19 species)				2 (11%)	6 (32%)	15 (79%)	18 (95%)	15 (79%)
*Danaus chrysippus*		16	85 (2% ± 2%)	2	1	13	–	Occurs
*Charaxes jasius*		2	Not analyzed	–	–	1	1	Occurs
*Limenitis reducta*		10	Not analyzed	–	–	7	3	Occurs
*Vanessa atalanta*		31	128 (2% ± 5%)	–	3	23	5	Occurs
*Vanessa cardui*		45	30,916 (65% ± 27%)	3	3	34	5	Occurs
*Polygonia egea*		2	Not analyzed	–	–	–	2	Occurs
*Melitaea ornata*		8	Not analyzed	–	1	6	1	Occurs
*Melitaea collina*		1	Not analyzed	–	–	–	1	–
*Melitaea trivia*		18	Not analyzed	–	1	15	3	Occurs
*Melitaea deserticola*		3	Not analyzed	–	1	1	1	–
*Melanargia titea*		26	453 (6% ± 6%)	–	–	20	5	–
*Ypthima asterope*		11	108 (2% ± 2%)	–	–	9	2	Occurs
*Hipparchia fatua*		11	196 (3% ± 4%)	–	–	9	1	Occurs
*Hipparchia pisidice*		3	Not analyzed	–	–	2	1	–
*Maniola telmessia*		28	919 (6% ± 10%)	–	–	23	5	Occurs
*Hyponephele lupina*		1	Not analyzed	–	–	–	1	Occurs
*Lasiommata megera*		8	Not analyzed	–	–	5	3	Occurs
*Lasiommata maera*		18	109 (1% ± 2%)	–	–	14	5	Occurs
*Kirinia roxelana*		2	Not analyzed	–	–	–	2	Occurs
Family: Lycaenidae (23 species)				9 (39%)	11 (48%)	16 (70%)	18 (78%)	17 (74%)
*Satyrium spini*		11	Not analyzed	–	–	9	2	Occurs
*Satyrium ilicis*		1	Not analyzed	–	–	–	1	Occurs
*Tomares nesimachus*		2	Not analyzed	–	–	2	–	–
*Deudorix livia*		13	Not analyzed	3	2	4	4	–
*Iolaus glaucus*		2	Not analyzed	2	–	–	–	–
*Apharitis acamas*		13	169 (2% ± 1%)	–	–	12	1	Occurs
*Apharitis cilissa*		1	Not analyzed	–	–	–	1	–
*Lycaena phlaeas*		14	99 (2% ± 2%)	–	–	9	5	Occurs
*Lycaena thersamon*		36	930 (4% ± 8%)	–	3	28	5	Occurs
*Lampides boeticus*		30	Not analyzed	2	2	21	5	Occurs
*Leptotes pirithous*		30	Not analyzed	1	2	22	5	Occurs
*Tarucus balkanicus*		6	Not analyzed	–	2	3	1	Occurs
*Tarucus rosaceus*		3	Not analyzed	2	1	–	–	–
*Azanus jesous*		15	2,708 (14% ± 28%)	2	3	8	2	Occurs
*Azanus ubaldus*		3	Not analyzed	2	1	–	–	Occurs
*Chilades galba*		4	Not analyzed	1	1	2	–	–
*Chilades trochylus*		23	874 (8% ± 12%)	–	1	18	4	Occurs
*Aricia agestis*		10	Not analyzed	–	–	5	5	Occurs
*Cyaniris semiargus*		1	Not analyzed	–	–	–	1	Occurs
*Polyommatus icarus*		28	916 (5% ± 6%)	–	–	23	5	Occurs
*Pseudophilotes vicrama*		23	Not analyzed	–	–	20	3	Occurs
*Glaucopsyche alexis*		1	Not analyzed	–	–	–	1	Occurs
*Zizeeria karsandra*		18	1,164 (12% ± 29%)	3	1	13	2	Occurs
Family: Hesperiidae (11 species)				0 (0%)	4 (36%)	9 (82%)	5 (45%)	11 (100%)
*Carcharodus alceae*		33	320 (2% ± 3%)	–	1	27	5	Occurs
*Carcharodus orientalis*		1	Not analyzed	–	–	1	–	Occurs
*Carcharodus stauderi*		2	Not analyzed	–	1	1	–	Occurs
*Spialia orbifer*		7	Not analyzed	–	–	6	1	Occurs
*Muschampia proteides stepporum*		1	Not analyzed	–	1	–	–	Different subspecies
*Syrichtus tessellum*		1	Not analyzed	–	–	–	1	Occurs
*Thymelicus acteon*		4	Not analyzed	–	–	4	–	Occurs
*Thymelicus sylvestris*		3	Not analyzed	–	–	3	–	Occurs
*Thymelicus hyrax*		7	Not analyzed	–	–	6	1	Occurs
*Pelopidas thrax*		11	Not analyzed	–	–	11	–	Occurs
*Gegenes pumilio*		11	Not analyzed	–	1	8	2	Occurs

^a^ Total species: total number of species found per ecoregion. Unique species: total number of species found in this ecoregion but not in others. Mean annual rainfall/temperatures: as calculated for the transects per ecoregion and not averaged over the entire ecoregion. NA: Not Applicable.

^b^ Occur. transects: number of transects in which the species occurred at least once in 5 years of monitoring (the environmental impacts on occurrence were not analyzed for species with fewer than four sites).

^c^ Abund.: total expected number of observations in adult butterflies in 2019 in all transects in the low Mediterranean ecoregion of Israel, if each transect was monitored once per week. *SD*: standard deviation.

^d^ Occurrence Transects: total number of transects analyzed for occurrence per ecoregion. High Mediterranean: >350 mm rainfall/annum, 700–1,300 m above sea level. Low Mediterranean: >350 mm rainfall/annum, <700 m above sea level. Semi‐desert: 200–350 mm rainfall/annum. Wadi Araba: 3 southernmost transects (Figure 1a); hyper‐arid (<50 mm rainfall per annum).

### 2.6 Analyzing species abundance

To estimate butterfly abundances despite differences in monitoring intensities and dates, we derived an abundance index which assesses the total number of adults that would have been recorded in each transect if it was visited weekly. However, since the BMS‐IL protocol suggests counting only twice per month, we used a phenological model to estimate how many butterflies would have been recorded in weeks when no counts were conducted. The abundance index is the sum of actual and imputed (modeled) counts. For more details, see Comay et al. (2020).

We used a regional generalized linear model (Regional GAM; Schmucki et al., 2016) to impute missing weekly counts using R version 3.6.2 (R Core Team, 2020), R Studio version 1.1.453 (RStudio Team, 2016), and rbms version 0.1.1 (Schmucki et al., 2019), with slightly modified functions (Appendix S2).

Regional GAM assumes each species flight season is identical in all input transects within a region. Many butterfly species have complex seasonal activity cycles, including multivoltine, aestivating, and migratory species (Comay et al., 2020). To derive reliable abundance estimates, we determined a minimum threshold of 100 visits for univoltine species with a single activity peak, and at least 200 visits for species with complex phenologies (for justification see Comay et al., 2020). We applied the threshold to all transects where relevant species occur. To limit reliance on imputed counts, we removed sites that were not visited during species' peak months. This procedure limited the analysis of species abundances to the low‐lying Mediterranean ecoregion, because other regions had too few data.

For the current analysis, we used abundance indices calculated based on sites that were visited at least six times during 2019 and at least six times between October 2018 and September 2019. In 2019, successful recruiting of volunteers enabled many new transects; therefore, we only analyzed the abundance of species we consider to be easily recognized by beginners. In sum, we retained a final species list comprising 24 species (Table 1) from 65 transects. After accounting for overlap with the 47 transects analyzed for occurrence, we analyzed a total of 88 transects (Appendix S1, Figure S1.1).

### 2.7 Species richness per ecoregion

We identified species found in each ecoregion and calculated their species richness. We compared the species list with a checklist of European butterfly species (Wiemers et al., 2018) to calculate the extent to which Israeli butterfly fauna is Palearctic in origin and to identify species that reach their global southern distribution edge in Israel. In addition, we compared species observed in BMS‐IL transects with those known from the entire study area (Israel Butterfly Monitoring Scheme, 2020), to identify how many species were expected in the study area but not found, and how their omission might influence the results.

### 2.8 Statistical analyses

We conducted analyses on the transect level (species list, richness, and abundance per transect) and on the species level, identifying the predictors that best explain species occurrence and abundance. We used negative binomial generalized linear models (function glm.nb in R package ‘MASS’; Venables & Ripley, 2002) to test how temperature, rainfall, soil, and vegetation affect species richness and total butterfly abundance (sum of all species' abundance indices) per transect. We selected the negative binomial distribution over the Poisson distribution because in both cases the variance was much higher than the mean. We selected models according to Akaike information criterion (AIC; Akaike, 1974) and Bayesian information criterion (BIC; Schwarz, 1978).

To analyze environmental affects, we fitted generalized linear latent variable models (GLLVM) to species occurrence (assuming a binomial distribution) and abundance (assuming a negative binomial distribution) data, using the R package ‘gllvm’ (Niku et al., 2019; Niku et al., 2020). Latent variables account for species covariances that are not accounted for by a model's predictors, such as interspecific interactions (e.g., competition, facilitation) or missing environmental predictors. When used without any predictors, latent variables describe the general species covariance pattern (i.e., species tending to occur at the same sites), regardless of the environment. Following the package authors' recommendations (Niku et al., 2019), we first assessed how the inclusion of one to five latent variables impacted the AIC, without any environmental predictors.

Next, we fitted a model with two latent variables and no explanatory variables, as a null model to compare the usefulness of adding covariates, as well as to draw an ordination plot of species and transects. In community ecology, an ordination plot depicts the similarity of two taxa or sites according to their distance. The closer the taxa are, the more likely they are to co‐occur; the closer the sites are, the more similar their faunas are. When the two are plotted together, the closer the taxon is to a site, the more likely it is to occur there. To plot the ordinations, we used two GLLVM models with two latent variables, one for occurrence and one for abundance. We plotted the 24 most indicative species (i.e., species differing the most between transects) according to the GLLVM in the species‐occurrence ordination and all 24 species in the species abundance ordination. We also used these GLLVM models to indicate species correlations following Niku et al. (2019). We plotted the correlation matrices using the R package ‘corrplot’ (Wei & Simko, 2017), and grouped species into four clusters for occurrence and four clusters for abundance using hierarchical clustering with complete linkages.

In addition, we used GLLVM to examine the statistical significance of the impact of four environmental variables (mean annual temperature and rainfall, habitat, and soil) on species' occurrence and abundance. We chose best‐fitting models according to AIC. Once models were selected, we plotted the statistically significant (*α* = 5%) species‐specific coefficients of their environmental predictors. We scaled the continuous predictors before fitting the models; therefore, coefficients were comparable in size. Notably, some soil and habitat types were found only in one or two transects; these were not included in the coefficient plot. We retained these transects in the GLLVM models, and their climatic data were included in the analysis.

## 3 RESULTS

### 3.1 Species richness per ecoregion

Table 1 details total species richness, number of unique species per ecoregion, species studied by taxonomic order, details of their occurrence in the four ecoregions and in Europe (Wiemers et al., 2018), and the total abundance index for all low‐lying Mediterranean sites. Species lists and abundance per transect, as well as the full GLLVM model results, are available in Dryad Digital Repository: https://doi.org/10.5061/dryad.2bvq83bqm.

Of 116 known species in the study area (Israel Butterfly Monitoring Scheme , 2020), 73 butterfly species were reported along the studied transects. Of the 43 missing species, two are new invasive alien species (*Chilades pandava* and *Cacyreus marshalli*) currently known from very restricted ranges, leaving 41 native ‘missing species’.

By and large, the Israeli butterfly fauna is Eurasian; 58 of the 73 (79%) species also occur in Europe (Wiemers et al., 2018; Table 1). Of the remaining 15 species, eight (53%) are restricted to the Middle East, six (40%) are distributed in Africa and southwest Asia, and one species (7%), *Melitaea deserticola*, is Saharo‐Arabian (Benyamini, 2010). A total of 36 Eurasian and Middle Eastern species reach their global southern edge of distribution in our study area, while only three African species reach their global northern edge of distribution therein.

On the country scale, Israeli butterfly fauna varies greatly with climate. Species richness decreases dramatically with aridity (Table 1). While 68 of the 73 species studied occur in Mediterranean ecoregions, only 29 reach the semidesert, and only 12 reach Wadi Araba. Wadi Araba has just 18 species, of which only two (*Colotis phisadia* and *Iolaus glaucus*) are unique. The semidesert ecoregion is also species poor (32 of 73), harboring a single unique subspecies (*Muschampia proteides stepporum*).

The inclusion of the ‘missing species’ (i.e., those that should occur in transects based on their distribution range) would strengthen the observed pattern: of the 43 ‘missing species’, 11 (26%) occur only in the High Mediterranean ecoregion, 2 (5%) are unique to the low‐lying Mediterranean ecoregion, 2 (5%) have been observed only along Wadi Araba and the Dead Sea, and none are expected to be unique to the semidesert ecoregion.

### 3.2 Common species

*Vanessa cardui*, *Colotis fausta*, *Pieris rapae*, and *Pieris brassicae* were the most widespread species in terms of occurrence, occurring in 41–45 out of the 47 transects studied (Table 1). Some species were abundant, but occurred in relatively few transects (e.g., *Zizeeria karsandra*, *Azanus jesous*). Others occurred in many transects but had medium or low abundance (e.g., *Colias crocea*, *Papilio machaon*, *Vanessa atalanta,* and *Carcharodus alceae*). *V. cardui* was the most abundant species; the next species in order of abundance, albeit an order of magnitude less abundant than *V*. *cardui*, were *C. fausta*, *P. brassicae*, and *P. rapae*.

### 3.3 Species richness and total butterfly abundance per transect

**FIGURE 2:**
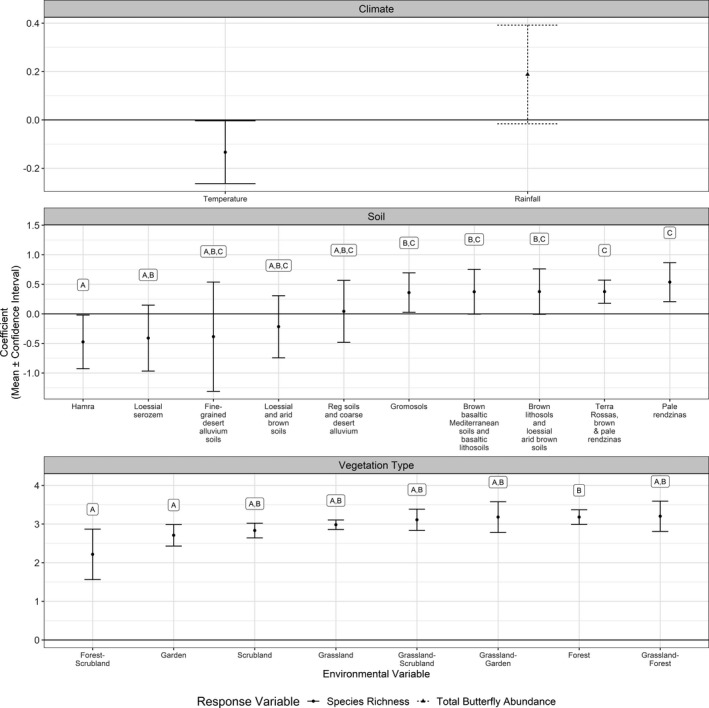
Coefficients of predictors of butterfly species richness for the entire study area (Israel, including the West Bank and Golan Heights) and total butterfly abundance in the low‐lying Mediterranean ecoregion, based on best‐fitting models (Appendix S1, Table S1.3). Higher coefficients indicate greater species richness, and vice versa. Overlapping confidence intervals (error bars) indicate nonsignificant differences. Categorical predictors (habitat and soil type, ordered from lowest to highest mean coefficient) sharing a letter label are not statistically significant

Figure 1a depicts species richness per transect, while Table S1.3 in Appendix S1 compares AIC and BIC between the generalized linear models of species richness and total butterfly abundance per transect. In the best‐fitting model, soil type, habitat (vegetation structure), and mean annual temperature significantly impacted species richness (Figure 2).

Species richness was higher for cooler transects. Transects on Hamra soils had fewer species than those on gromosols, basaltic soils, Terra rossa, rendzines, and lithosols. In addition, transects on loessial sierozem had fewer species than those on Terra rossas and pale rendzines. Fewer species were found in forest‐scrubland mixtures and in gardens (panel (a) in Figure 2) compared with forest‐only transects (panel (b) in Figure 2). Grasslands, scrubland, and grasslands mixed with other habitats were ranked A and B (i.e., between the two levels) but also had notably higher variance compared with forest transects.

Figure 1b depicts total butterfly abundance per transect. In the low‐lying Mediterranean region, rainfall was the only predictor in the best‐fitting model (i.e., lowest AIC and BIC; Appendix S1 Table S1.3), with a marginally insignificant (*p*‐value = .071) positive impact (i.e., more butterflies along transects with higher precipitation). Although a total abundance model using only temperature had similar AIC and BIC values (Table S5), a likelihood ratio test comparing it with a model using only rainfall found a significant difference (*p*‐value < 10^–3^), indicating that the rainfall‐only model had a better fit.

### 3.4 Community composition

**FIGURE 3:**
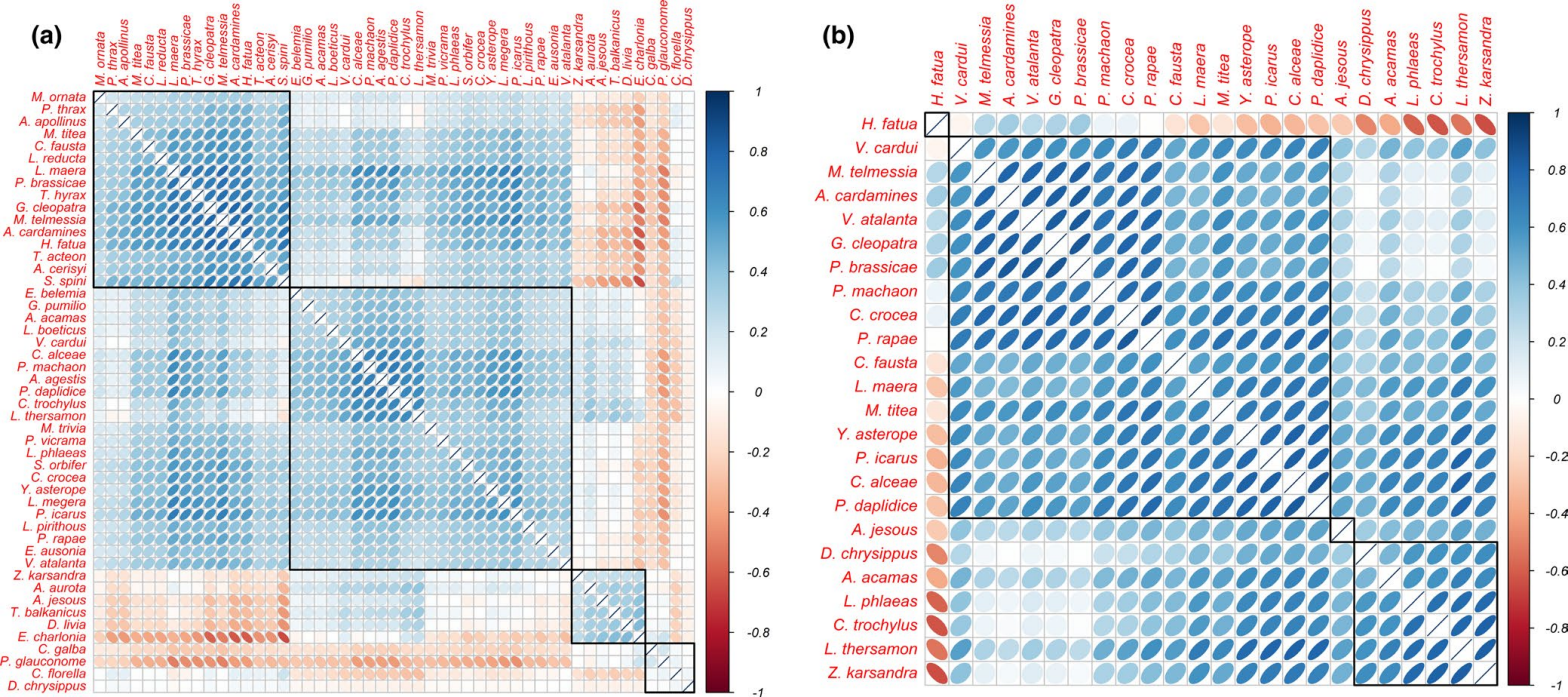
Interspecific correlations based on Israeli butterfly species occurrence in the entire study area (a) and abundance in the low‐lying Mediterranean ecoregion alone (b). Red ellipses are negative correlations and blue ellipses are positive correlations. Correlations were extracted from generalized linear latent variable models without using any predictors (see Methods for details). Black squares denote the four species clusters created by the hierarchical clustering algorithm with complete linkages

Figure 1c depicts the ordination of both transects and species by occurrence compared with the geographic locality of transects (Figure 1a); axes are the latent variables of the GLLVM model fitted without any predictors (e.g., temperature, habitat), and hence, they represent overall trends in the data (i.e., similarity and dissimilarity between transects or species in terms of occurrence) in two dimensions. Figure 1d repeats the results for abundance. Figure 3 depicts correlations among species (i.e., how much they overlap with each other regardless of environmental predictors) based on occurrence (Figure 3a) in the entire study area and abundance in the low‐lying Mediterranean ecoregion (Figure 3b).

The three hottest transects (red numbers), located in Wadi Araba, can be seen on the left side of Figure 1c, while all cool transects (light blue) are on the right. When examining occurrence, only one species out of 24, *Euchloe charlonia*, had negative correlations with many other species, and especially with *Satyrium spini* (Figure 3a). This strong negative correlation is in accordance with the species' distinct Saharan distribution covering the eastern and southern parts of the study area (Benyamini, 2010), overlapping that of most other species only around Jerusalem. Most grassland transects are on the lower part of the ordination plot (i.e., negative values in the second latent variable), while most scrubland and forest transects are on the upper part (i.e., positive values in the second latent variables). In the upper right quarter of Figure 1c, a cluster of species (*Satyrium spini*, *Anthocharis cardamines*, *Hipparchia fatua*, *Gonepteryx cleopatra*, *Maniola telmessia,* and *Thymelicus hyrax*) are all positively correlated with each other (Figure 3a). In the lower right quarter, *Polyommatus icarus*, *Lasiommata megera*, *Yphtima asterope*, *Lycaena phlaeas*, *Colias crocea*, and *Pseudophilotes vicrama* cluster together and often co‐occur (Figure 3a). Another cluster, lower in the same quarter, comprises *Papilio machaon*, *Pontia daplidice*, *Lycaena thersamon*, *Chilades trochylus*, and *Aricia agestis*. These species all occur in Mediterranean Israel and some in its semidesert ecoregion, but not in the hottest and arid areas (i.e., Wadi Araba; Table 1). Close to this cluster are *Vanessa cardui* and *Carcharodus alcaea* (both generalists and widespread throughout the country).

The ordination of the transects and butterfly fauna in terms of species abundances (Figure 1d) shows that *Hipparchia fatua* (a forest species) stands out from the other species (and has negative correlations with many species; Figure 3b), while *M. telmessia*, *A. cardamines*, *P. brassicae*, *G. cleopatra*, and *V. atalanta* cluster in the lower right quarter and are all positively correlated to one another (Figure 3b). These species can be characterized by a Mediterranean distribution and an affiliation to heterogeneous habitats (mix of trees, scrub, and grass; see also Schultz et al., 2017). In the upper right corner, the cluster of *Zizeeria karsandra*, *Chilades trochylus*, *Lycaena phlaeas,* and *L*. *thersamon* represents species often occurring in disturbed habitats. Members of each of these clusters have very weak correlations with members of the other cluster (Figure 3b), indicating clusters' independence from one another. Several migratory species (*V*. *cardui*, *C. fausta*, *Danaus chrysippus*, and *A. jesous*) are in the upper left part of the ordination, but do not form a clear cluster. Moreover, other migrant species occur elsewhere in the ordination (*V*. *atalanta, P. daplidice*, and *C*. *crocea*), but are positively correlated with many other species (Figure 3b).

### 3.5 Factors shaping species occurrence and abundance

**FIGURE 4:**
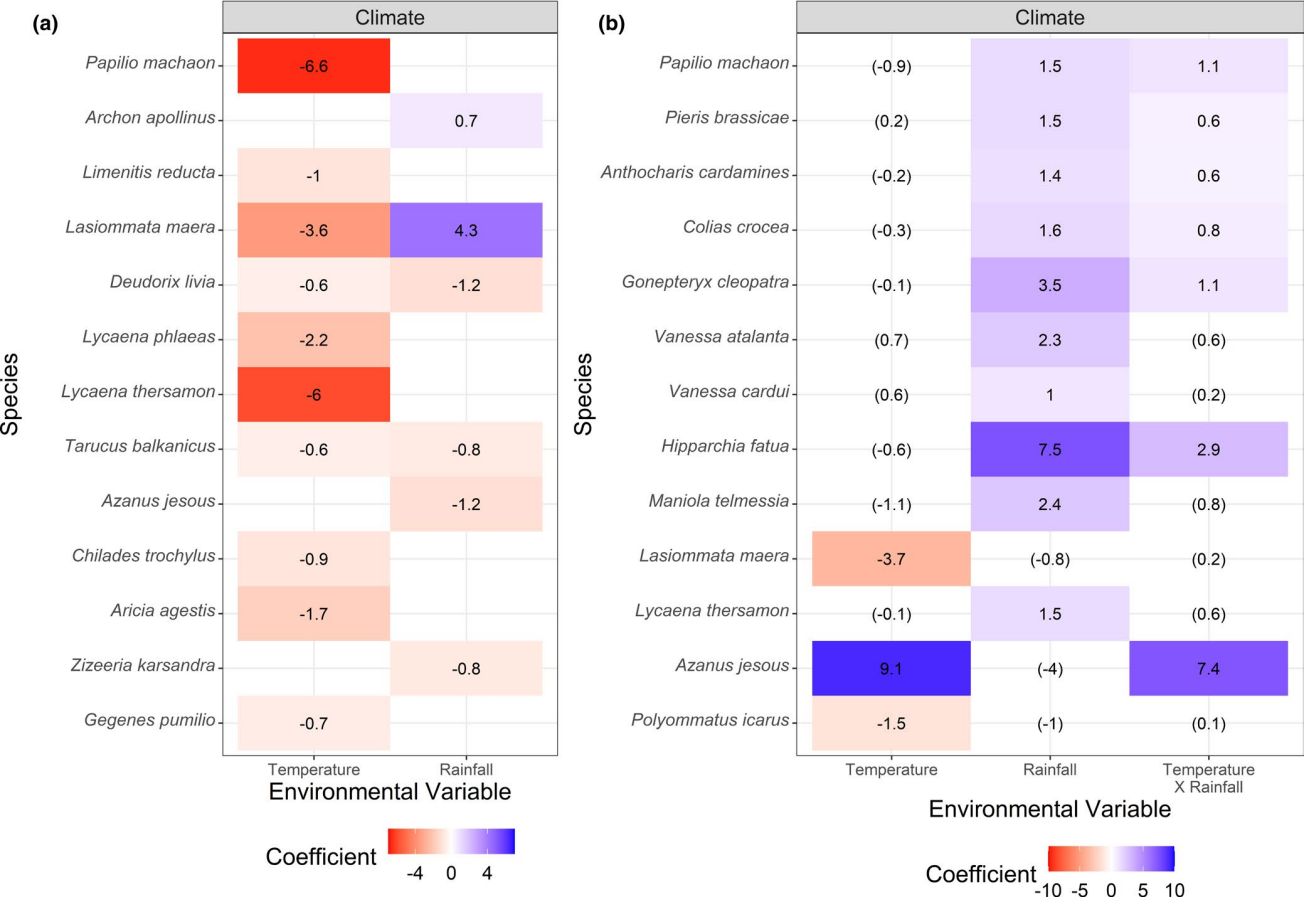
Coefficients of significant predictors of butterfly species occurrence for the entire study area (Israel, including the West Bank and Golan Heights); (a) and abundance within the low Mediterranean region (b). Species with no significant predictors are not depicted. Positive coefficients indicate species that tend to occur (or be more abundant) when the value of the predictor is higher (e.g., warmer temperature or more rainfall) or when a particular category of the predictor (e.g., soil type) is present, and vice versa. Shading depicts the strength of deviation (blue = positive, red = negative), assigning colors only to significant results. In (b), insignificant predictors are given in brackets and are not colored; they should be used to interpret the interaction term

Figure 4 depicts the significant coefficients of occurrence (Figure 4a, in the entire study area) and abundance (Figure 4b, only within the low‐lying Mediterranean region). The best‐fitting (lowest AIC) GLLVM models of both occurrence and abundance had significant coefficients for temperature and rainfall, but not for habitat or soil type. The latter are therefore not depicted in Figure 4.

Temperature had either a negative or an insignificant impact on species occurrence (i.e., species were either less likely to occur along warmer transects, or were unaffected by temperature; Figure 4a). However, within the low‐lying Mediterranean region (Figure 4b), temperature had a significant impact on the abundance of just three species, two negatively (*Lasiommata maera, Polyommatus icarus*) and one positively (*A. jesous*), confirming the latter as a warmth‐tolerant species in accordance with its Afrotropical distribution (Benyamini, 2010). Rainfall was found to significantly explain the occurrence of six species (four negatively, two positively), but had a significant impact on abundance for ten species (all positively). The interaction between temperature and rainfall was found to be significant for seven species. Only two species that had significant predictors of abundance were not affected by rainfall or by its interaction with temperature (*L. maera* and *P*. *icarus*); both were less abundant along warmer transects.

## 4 DISCUSSION

Overall, we observed a clear trend of decreasing species richness from cooler to warmer ecoregions, with fewer Palearctic species occurring under increasingly arid conditions; this is consistent with previous studies (e.g., Benyamini, 2010; Pe'er et al., 2011). In contrast, based on an extensive review, Hawkins et al. (2003) concluded that water availability should be the strongest predictor of species richness (in general and specifically for butterflies) south of ~45°N. In other words, our results deviate from the general pattern of species richness drivers at ~30°N because temperature was the strongest predictor of species richness.

The observed decrease in species richness with temperature (Table 1) also contradicts a global trend of increasing butterfly species richness at lower latitudes (Hawkins, 2010). Nevertheless, closer examination of global and European patterns reveals higher butterfly species richness in mountainous areas (Hawkins, 2010). In other words, the pattern observed in our data is similar to the general trend observed in southern Europe, with higher species richness in mountainous (and thus cooler) areas. These results reflect the fact that there are far fewer species of African origin, and hence, their presence in the warmer south does not compensate (in terms of species richness) for the loss of Palearctic species.

In addition to the effect of temperature, we found that habitat and soil also affect butterfly species richness (Figure 2), while rainfall by itself is insignificant. However, total butterfly abundance was impacted only by rainfall (Figure 2), with a positive correlation for most species (Figure 4b). As soil and vegetation depend on climate, it is likely that plant community composition, rather than habitat architecture, might be the mechanism driving butterfly species richness. Vegetation, described here using general categories, is in fact more accurately described by soil type; that is, under similar climate conditions, different floras will develop in the poor coastal soils (Hamra and sand dunes; Singer, 2007) and in rendzinas and Terra rossas (Dan, 1991). Moreover, Schwartz‐Tzachor (2007) found a significant correlation between nectar plant species richness and butterfly species richness in Israel. Therefore, butterfly species richness can vary spatially according to micro‐environmental conditions.

Our results (that climate has a stronger impact on butterfly biodiversity than land cover) are in line with the patterns observed elsewhere. Kivinen et al. (2007) found that in Finland, climate explains overall butterfly species richness better than land cover. In Egypt, both climate and habitat significantly affected butterfly species richness (Newbold et al., 2009). Likewise, Gutiérrez Illán et al. (2010) found that topoclimatic models better explain patterns than do land‐cover models. Stefanescu et al. (2004) found a strong climatic impact on butterfly species richness compared with a weak impact of vegetation structure.

Temperature is the dominant factor dictating occurrence, with many species associated with cooler climates (Figure 4a). The lack of species that are clearly associated with warmer temperatures may indicate that species that appear to be warmth‐oriented are rather robust to heat, that is, occur both in cooler and warmer climates. Alternatively, this could be an artifact of the low number of transects in the hyper‐arid Wadi Araba and semidesert, where typical Saharan or sub‐Saharan species occur. Nevertheless, we did identify four species with higher occurrence in arid areas (Figure 4a), and one showing significantly higher abundance at higher temperature (*A. jesous*). Benyamini and Müller (2020) suggested that climate change will increase the frequency of cyclones and of the African easterly jet, which in turn will facilitate the introduction and establishment of Afrotropical butterflies in the Middle East. If these introductions of Afrotropical species continue, it could enrich the species‐poor fauna of Wadi Araba.

Rainfall was a good predictor of total butterfly abundance, while temperature was not (Figure 2). Therefore, our results offer a more refined picture of the potentially divergent impacts of changes in temperature versus precipitation under climate change. Our biogeographical evaluation indicates more losers than winners from increased temperature. This is in line with the high species diversity at high altitudes (Table 1), making Israel's mountains, as with other mountain regions of the world (Spehn et al., 2010), biodiversity hotspots. The projections and risks to species needing to ascend in altitude have also been shown elsewhere (Freeman et al., 2018). Under the projected shift to shorter, warmer winters and higher frequency drought conditions (Hochman et al., 2018), one may anticipate not only poleward and upward shifts in distributions, but also an overall fall in abundance. In the context of insect decline, this portrays climate change as a significant source of pressure on butterfly fauna in the region.

### 4.1 Butterflies as bioindicators in Israel

We found that vegetation and soil were not good predictors of occurrence or abundance (Figure 2), at least on the scale and resolution used in this study. In contrast, climate, and especially temperature, had a strong impact on butterfly fauna; furthermore, butterflies respond more rapidly to climate change than birds (Devictor et al., 2012). This confirms that butterflies could be used as bioindicators for climate change impact on fauna.

While butterflies could also potentially serve as habitat bioindicators in Israel, our results do not offer conclusive affiliations of species with habitats per se. Based on our results alongside expert knowledge and other studies (e.g., Pe'er et al., 2011; Schultz et al., 2017; Schwartz‐Tzachor, 2007), we propose the potential for five climate and habitat indicator types (Table 2): (a) warmth‐intolerant, (b) warmth‐tolerant, and species occurring in (c) Mediterranean natural habitats, (d) disturbed Mediterranean habitats, and (e) on the coastal plain, including African migrants not reaching higher altitudes or latitudes. The latter two groups require further research. See Appendix S3 for further discussion.

**TABLE 2:** Bioindication potential of butterfly species in Israel^a^

Group	Species	References
Warmth‐intolerant (cool microclimate indicators)	*Papilio machaon*	Figure 4
	*Lasiommata maera*	
	*Lycaena thersamon*	
	*Lycaena phlaeas*	
	*Aricia agestis*	
	*Polyommatus icarus*	
Warmth‐tolerant (warm microclimate indicators)	*Azanus jesous*	Figure 3a and Figure 4b
	*Deudorix livia*	
	*Tarucus balkanicus*	
	*Zizeeria karsandra*	
	*Euchloe charlonia*	
	*Anaphaeis aurota*	
Mediterranean natural habitats (scrublands, maquis, and forests)	*Archon apollinus*	Figure 3a, Schultz et al. (2017)
	*Allancastria cerisyi*	
	*Pieris brassicae*	
	*Colotis fausta*	
	*Anthocharis cardamines*	
	*Gonepteryx cleopatra*	
	*Hipparchia fatua*	
	*Lasiommata maera*	
	*Limenitis reducta*	
	*Melitaea ornata*	
	*Maniola telmessia*	
	*Melanargia titea*	
	*Pelopidas thrax*	
	*Satyrium spini*	
	*Thymelicus acteon*	
	*Thymelicus hyrax*	
Species occurring in Mediterranean disturbed habitats, including agricultural and fallow fields, roads, archaeological sites, or gardens. Including species with agricultural, cultivated, or nitrophilous host plants	*Apharitis acamas*	Figure 3a, Schwartz‐Tzachor (2007), Schultz et al. (2017), expert knowledge
	*Aricia agestis*	
	*Carcharodus alceae*	
	*Colias crocea*	
	*Chilades trochylus*	
	*Euchloe ausonia*	
	*Euchloe belemia*	
	*Gegenes pumilio*	
	*Lampides boeticus*	
	*Lasiommata megera*	
	*Lycaena phlaeas*	
	*Leptotes pirithous*	
	*Lycaena thersamon*	
	*Melitaea trivia*	
	*Pontia daplidice*	
	*Polyommatus icarus*	
	*Papilio machaon*	
	*Pieris rapae*	
	*Pseudophilotes vicrama*	
	*Spialia orbifer*	
	*Vanessa atalanta*	
	*Vanessa cardui*	
	*Ypthima asterope*	
Coastal plain species, including African migrants not reaching higher altitudes or latitudes	*Catopsilia florella*	Figure 3a, expert knowledge
	*Chilades galba*	
	*Danaus chrysippus*	
	*Pontia glauconome*	

^a^ Note that some species (*Papilio machaon*, *Lycaena thersamon*, *L*. *phlaeas,* and *Polyommatus icarus*) appear in more than one group.

### 4.2 Limitations and outlook

The uneven distribution of active transects generates a biased representation of biogeographical regions and habitats (Figure 1a, Table 1), with better coverage of the low‐lying Mediterranean region. This is the only region where data were sufficient to reliably assess both occurrence and abundance (Comay et al., 2020). Transects are rare in desert and semiarid parts of the country, together covering 58% of the study area. The arid but cool Negev Mountains, as well as the alpine Mt. Hermon, are currently not covered by any transects (Figure 1a). Efforts are underway to expand butterfly monitoring into these regions.

This uneven transect distribution is a limiting factor in the analysis of species' habitat affiliations. Notably, bias in terms of habitats is also due to volunteers' preference of natural and seminatural habitats, or nearby sites such as parks. Transects are completely missing on agricultural land. While targeted recruitment of volunteers would help to address such biases, the use of paid observers for targeted regions and habitats may be inevitable for closing key gaps. In addition, the highly variable total abundances within nearby transects (Figure 1b) and the significant impacts of habitat or soil type (Figure 2) suggest that more transects are needed to understand local drivers of butterfly biodiversity.

Another limitation is our reliance on single‐year (2019) abundance data, rather than a summary (e.g., mean or median) of several years. This was done in order to include as many transects as possible and to better represent climatic and habitat variability within the low‐lying Mediterranean ecoregion. In addition, BMS‐IL has grown rapidly in recent years (Comay et al., 2020); thus, 2019 represents both the most recent data and also the year with the best geographical representation of the country. Future data, especially after one or more drought years, could result in considerably different abundance data, such as fewer *V*. *cardui* butterflies, which dominated all transects in 2019 (Table 1). Nevertheless, given the overall agreement between prior expert assessments of species abundance (Benyamini, 2010) and our own results, it is unlikely that the general relationships among environmental variables, abundance, and occurrence will change dramatically.

A final limitation of our study is the reliance on exactly five years of data for compiling species lists. Some of the species described as missing from biogeographical regions (Table 1) would be included if a larger number of years were analyzed. This stresses the importance of preserving numerous transects for long periods, in order to ensure representative species lists over a large geographical extent.

## CONFLICT OF INTEREST

The authors declare no conflict of interest.

## AUTHOR CONTRIBUTIONS

**Orr Comay**: Conceptualization (lead); formal analysis (lead); methodology (lead); visualization (lead); writing‐original draft (lead); writing—review and editing (lead). **Oz Ben Yehuda**: Project administration (supporting); validation (lead); writing—review and editing (equal). **Racheli Schwartz‐Tzachor**: Funding acquisition (equal); investigation (supporting); project administration (lead); writing—review and editing (supporting). **Dubi Benyamini**: Funding acquisition (supporting); project administration (equal); writing—review and editing (supporting). **Israel Pe'er**: Data curation (lead); funding acquisition (equal); investigation (supporting); project administration (equal); software (lead); writing—review and editing (equal). **Inbar Ktalav**: Investigation (supporting); project administration (supporting); writing—review and editing (equal). **Guy Pe'er**: Conceptualization (equal); funding acquisition (lead); project administration (equal); supervision (lead); writing—review and editing (equal).

## Supporting information

Appendices S1 and S3

Appendix S2

## Data Availability

Species occurrence and abundance, as well as transects locations and environmental variables (mean annual temperature and precipitation, habitat), can be accessed through Dryad Digital Repository https://doi.org/10.5061/dryad.2bvq83bqm.
