# Supplementary material for: Environmental controls on butterfly occurrence and species richness in Israel: The importance of temperature over rainfall
Source: Ecol Evol. 2021 Aug 2;11(17):12035–50. doi: 10.1002/ece3.7969 (PMC8427576; doi:10.1002/ece3.7969)
Supplement: Supplementary file 1 — Appendices S1 and S3 [file ECE3-11-12035-s002.docx]

**Appendix S1: Supplementary figures and tables**

**Supplementary figures**

**
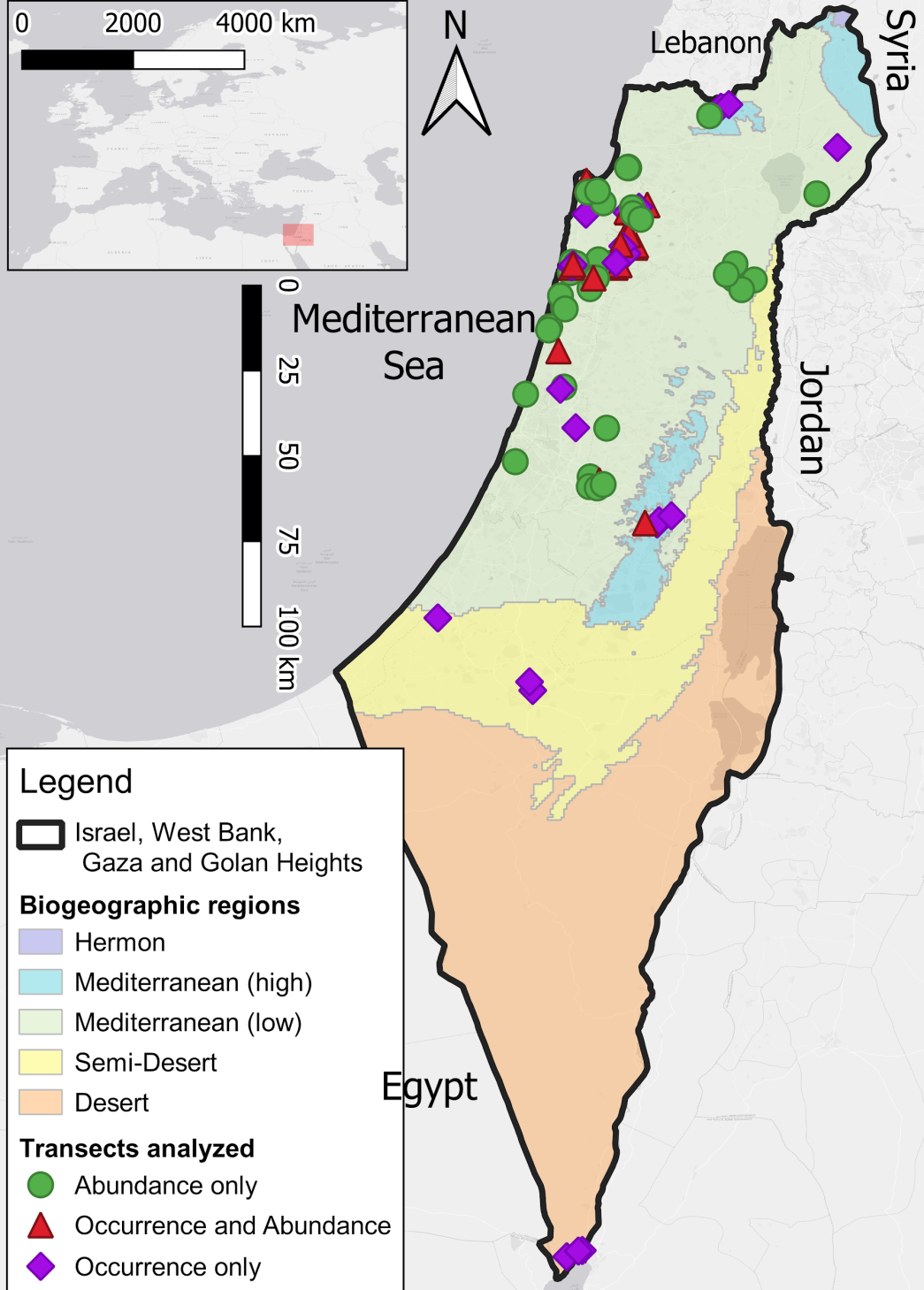
**

**Figure S1.1.** Ecoregions of the study area and the 88 Pollard transects analysed. Desert: < 200 mm rainfall/annum; Semi-desert: 200–350 mm rainfall/annum; Mediterranean (low): > 350 mm rainfall/annum, > 700 m elevation; Mediterranean (high): > 350 mm rainfall/annum, 700–1300 m elevation; Mt. Hermon: > 1300 mm rainfall/annum (no transects).


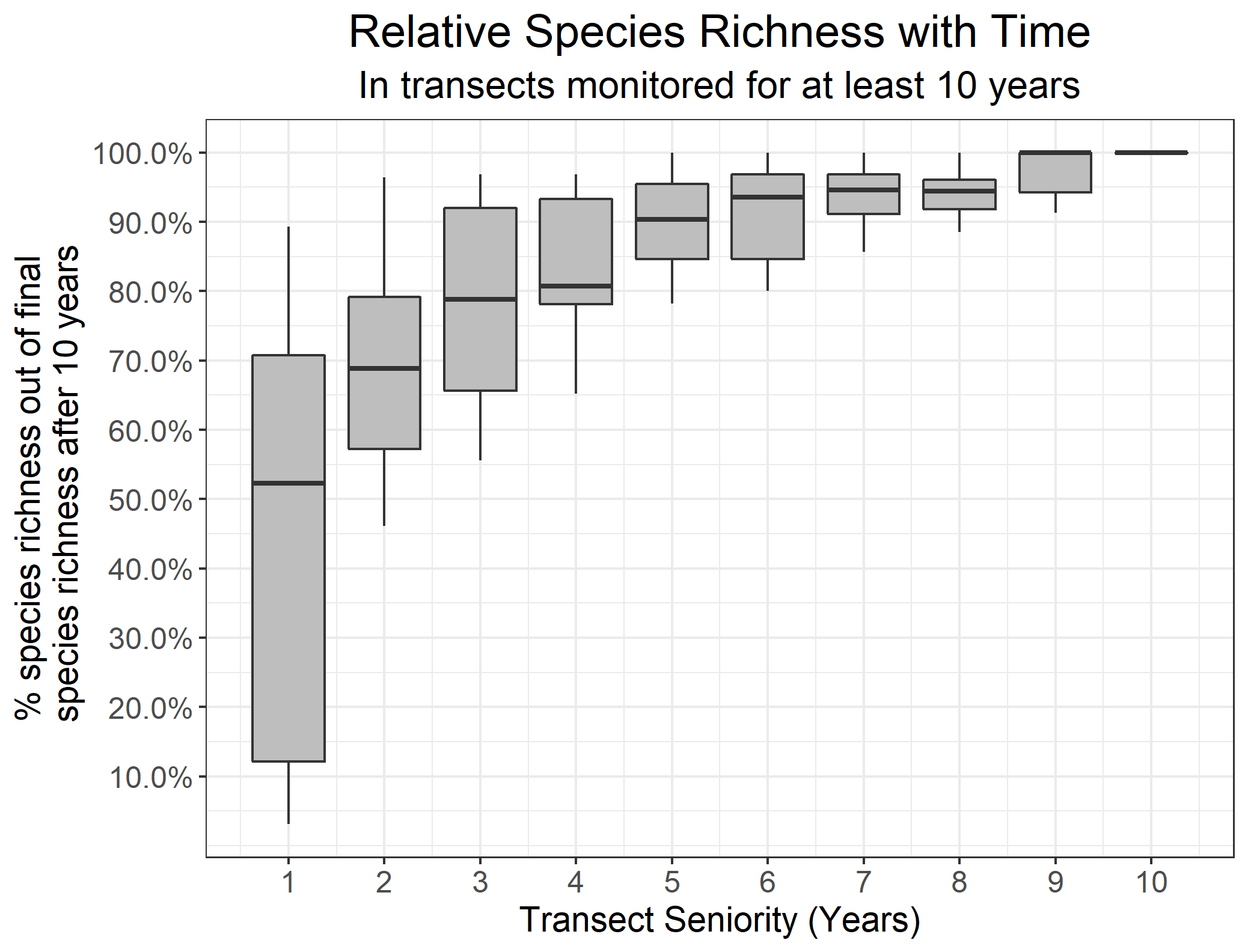


**Figure S1.2.** Boxplots of relative Israeli butterfly species richness per transect in 10 consecutive monitoring years. A value of 100% is the final species richness after 10 monitoring years in the specific transect (the exact value varies between transects). N = 14 transects.


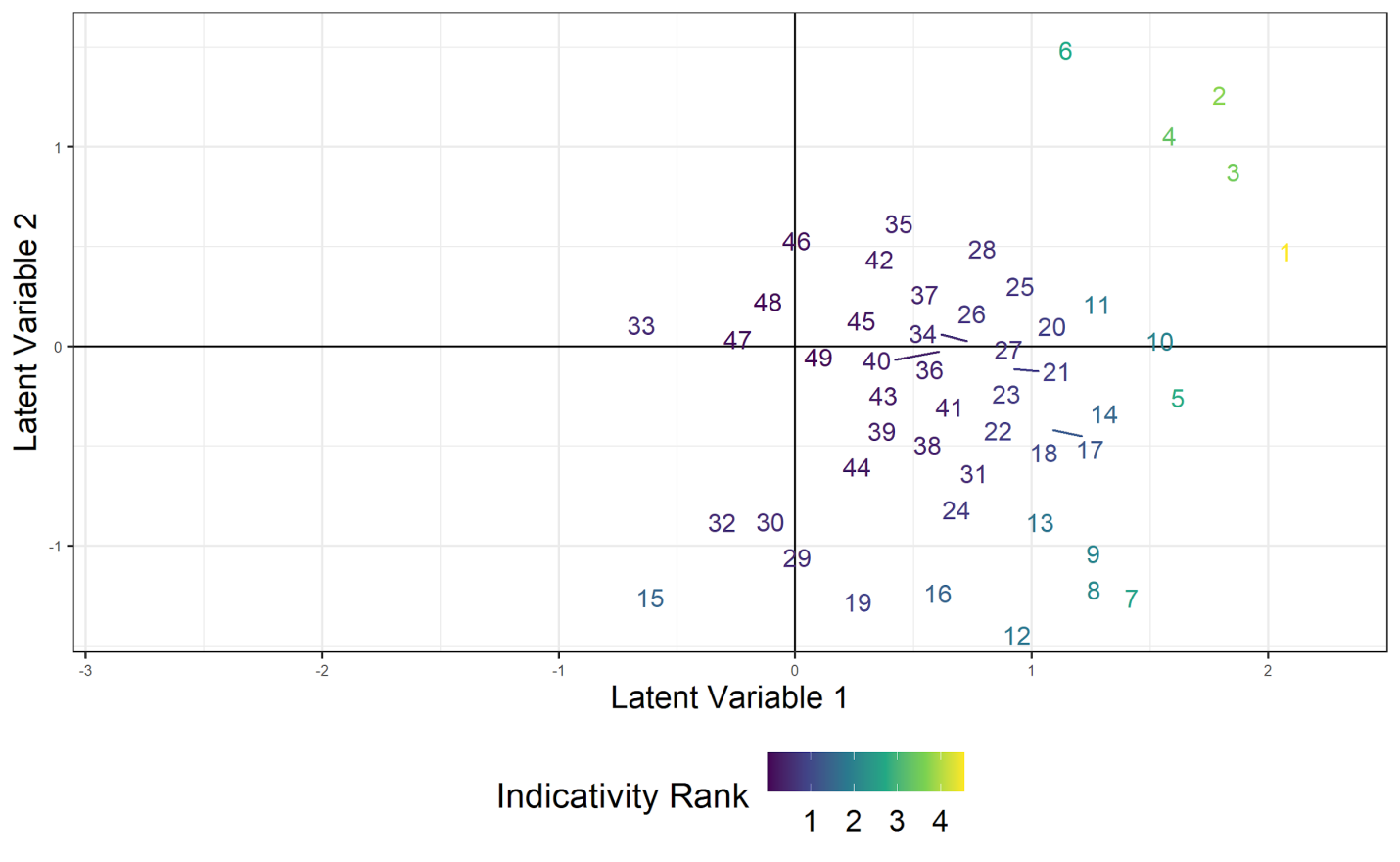


**Figure S1.3.** Generalised linear latent variable model (GLLVM)-based ordination of Israeli butterfly species by occurrence/absence. Species are ranked by indicativity (i.e., how much they differentiate the transects). 1) *Maniola telmessia*; 2) *Anthocharis cardamines*; 3) *Gonepteryx cleopatra*; 4) *Hipparchia fatua*; 5) *Lasiommata maera*; 6) *Satyrium spini*; 7) *Pontia daplidice*; 8) *Aricia agestis*; 9) *Papilio machaon*; 10) *Pieris brassicae*; 11) *Thymelicus hyrax*; 12) *Chilades trochylus*; 13) *Carcharodus alceae*; 14) *Polyommatus icarus*; 15) *Euchloe charlonia*; 16) *Lycaena thersamon*; 17) *Lasiommata megera*; 18) *Ypthima asterope*; 19) *Anaphaeis aurota*; 20) *Melanargia titea*; 21) *Colias crocea*; 22) *Lycaena phlaeas*; 23) *Pseudophilotes vicrama*; 24) *Vanessa cardui*; 25) *Limenitis reducta*; 26) *Colotis fausta*; 27) *Spialia orbifer*; 28) *Thymelicus acteon*; 29) *Tarucus balkanicus*; 30) *Deudorix livia*; 31) *Lampides boeticus*; 32) *Azanus jesous*; 33) *Pontia glauconome*; 34) *Pieris rapae*; 35) *Archon apollinus*; 36) *Leptotes pirithous*; 37) *Vanessa atalanta*; 38) *Apharitis acamas*; 39) *Gegenes pumilio*; 40) *Euchloe ausonia*; 41) *Melitaea trivia*; 42) *Pelopidas thrax*; 43) *Euchloe belemia*; 44) *Zizeeria karsandra*; 45) *Melitaea ornata*; 46) *Catopsilia florella*; 47) *Chilades galba*; 48) *Danaus chrysippus*; 49) *Allancastria cerisyi*.


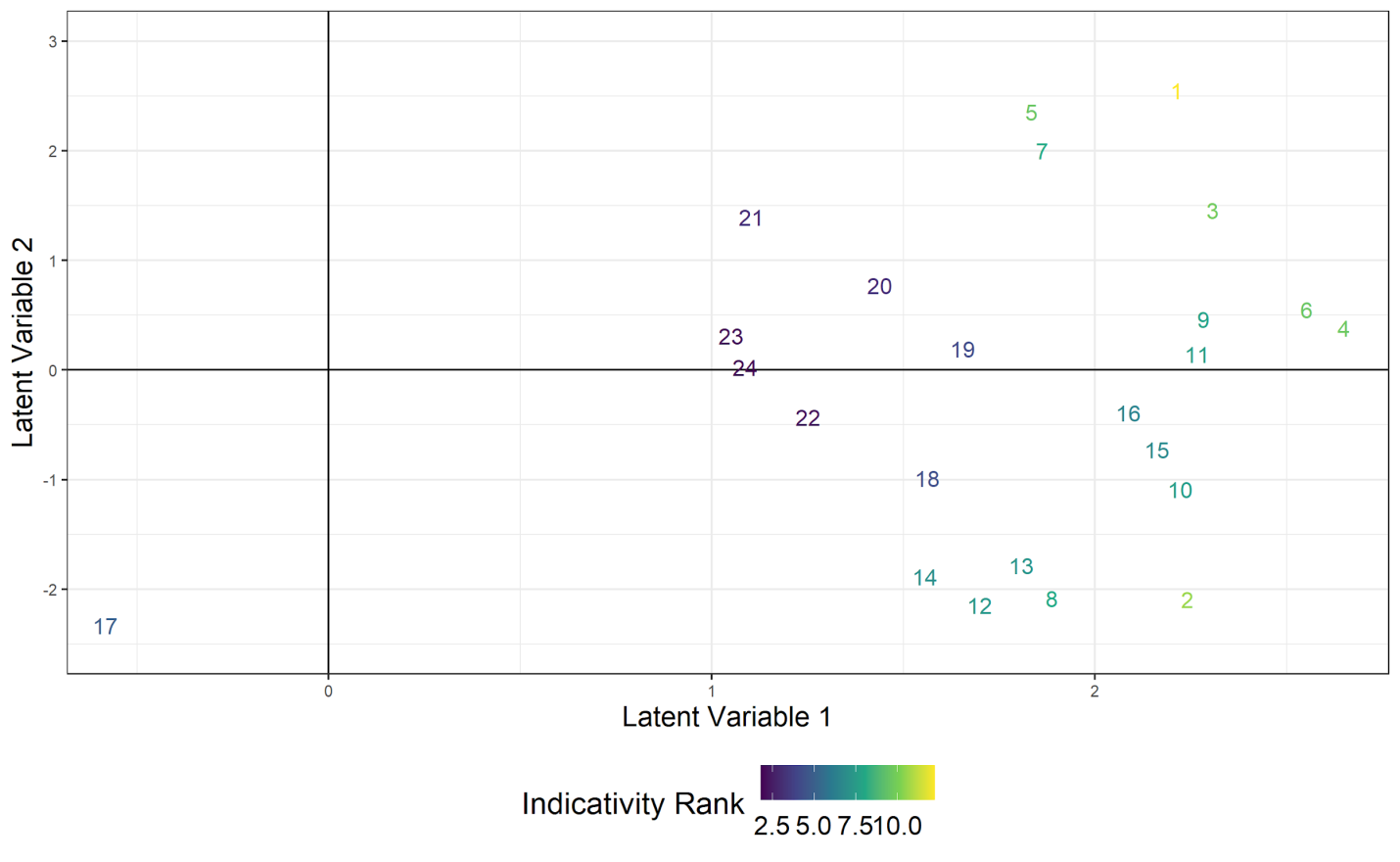
**Figure S1.4.** Generalised linear latent variable model (GLLVM)-based ordination of Israeli butterfly species by abundance. Species are ranked by indicativity (i.e., how much they differentiate the transects). 1) *Zizeeria karsandra*; 2) *Vanessa atalanta*; 3) *Lycaena thersamon*; 4) *Ypthima asterope*; 5) *Chilades trochylus*; 6) *Carcharodus alceae*; 7) *Lycaena phlaeas*; 8) *Gonepteryx cleopatra*; 9) *Polyommatus icarus*; 10) *Colias crocea*; 11) *Pontia daplidice*; 12) *Pieris brassicae*; 13) *Maniola telmessia*; 14) *Anthocharis cardamines*; 15) *Pieris rapae*; 16) *Melanargia titea*; 17) *Hipparchia fatua*; 18) *Papilio machaon*; 19) *Lasiommata maera*; 20) *Apharitis acamas*; 21) *Danaus chrysippus*; 22) *Vanessa cardui*; 23) *Azanus jesous*; 24) *Colotis fausta*.

**Supplementary tables**

**Table S1.1.** Climate, transects, and butterfly species per ecoregion in Israel

| Region^a^ | Mean Annual Rainfall (mm)^b^ | Mean Annual Temperature (°C)^b^ | Occurrence Transects | Species richness | Unique species^c^ |
| --- | --- | --- | --- | --- | --- |
| All transects combined | 529 ± 172 | 19.0 ± 1.3 | 47 | 73 | NA |
| High Mediterranean | 589 ± 203 | 16.6 ± 0.3 | 5 | 57 | 11 |
| Low-lying Mediterranean | 585 ± 47 | 19.0 ± 0.4 | 36 | 56 | 6 |
| Semi-desert | 277 ± 60 | 19.2 ± 0.3 | 3 | 32 | 1 |
| Wadi Araba | 15 ± 1 | 22.8 ± 0.1 | 3 | 18 | 2 |

^a^High Mediterranean: > 350 mm rainfall/annum, 700–1300 m above sea level; Low-lying Mediterranean: > 350 mm rainfall/annum, < 700 m above sea level; Semi-desert: 200–350 mm rainfall/annum; Wadi Araba: 3 southernmost transects (Fig. 1); hyper-arid (< 50 mm rainfall per annum).

^b^Climate data are presented as the mean ± standard deviation of transects whose species lists were studied, and not of the entire region.

^c^Unique species are those occurring in the ecoregion and in no other. NA = Not Applicable.

**Table S1.2.** Dominant soil types in Israel (including West Bank and Golan Heights), summarised after Singer (2007) and Dan (1991).

| Soil type | Region (ecoregion in this study) | Fertility | Climax Vegetation (dominant species) |
| --- | --- | --- | --- |
| Rendzinas and Terra Rossa | Mediterranean hills and mountains (low-lying and high Mediterranean) | High | Mediterranean maquis or open oak forest (*Quercus* *calliprinos* - *Pistacia terebinthus*) |
| Basaltic protogromsols | Eastern Lower Galilee and southern Golan Heights (low-lying Mediterranean) | Low | Open warmth-adapted forest or scrubland (*Quercus ithaburensis* or *Ziziphus* spp.) |
| Grumusol | Valleys in the Mediterranean region (low-lying Mediterranean) | Moderate | Lost to extensive cultivation; possibly semi-arid open scrubland |
| Hamra | Coastal Plain (low-lying Mediterranean) | Low | Open warmth-adapted oak forest (*Quercus ithaburensis*) |
| Serozem | Jordan Valley (low-lying Mediterranean and semi-desert) and northwestern Negev (semi-desert) | High | Aridity-adapted open scrubland (*Haloxylon scoparium* - *Anabasis syriaca*) |
| Loess | Northern Negev (semi-desert) | High | Aridity-adapted open scrubland (*Haloxylon scoparium* - *Anabasis syriaca*) |
| Dunes | Coastal Plain (low-lying Mediterranean); Northwestern Negev (Semi-desert) | Low | Psammophilous grassland (*Artemisia monosperma* - *Cyperus macrorrhizus*) |
| Reg and desert alluvium | Wadi Araba (desert) | Very low | Aridity-adapted open scrubland in stream beds (*Anabasis articulata*) |

**Table S1.3.** Generalized linear model selection for Israeli butterfly species richness and total butterfly abundance per transect using Akaike (AIC) and Bayesian (BIC) criteria^a^

| **Model no.** | **Habitat** | **Soil** | **Temp.^b^** | **Rain^c^** | **Species Richness** | | **Total Butterfly Abundance^d^** | |
| --- | --- | --- | --- | --- | --- | --- | --- | --- |
|  |  |  |  |  | **ΔAIC^e^** | **ΔBIC^e^** | **ΔAIC^e^** | **ΔBIC^e^** |
| **4** | **V** | **V** | **V** | **-** | **0** | 20.4 | 16.8 | 45.1 |
| **1** | **V** | **V** | **V** | **V** | **1.8** | 24.1 | 18.8 | 49.3 |
| 3 | V | V | - | V | 3.9 | 24.3 | 17.7 | 46.0 |
| 7 | - | V | - | - | 6.1 | 11.7 | 10.3 | 23.4 |
| 6 | - | V | V | - | 6.6 | 14.1 | 10.2 | 25.5 |
| 13 | - | V | - | V | 8.1 | 15.5 | 10.5 | 25.7 |
| 5 | - | V | V | V | 8.6 | 17.9 | 11.9 | 29.3 |
| 12 | V | - | V | - | 10.5 | 12.4 | 7.6 | 20.7 |
| **9** | **-** | **-** | **V** | **-** | 11.0 | **0** | **0.7** | **0.7** |
| 2 | V | - | V | V | 12.5 | 16.3 | 9.1 | 24.4 |
| **11** | **-** | **-** | **V** | **V** | 12.8 | 3.7 | **1.6** | 3.8 |
| 15 | - | - | Temp., Rain & interaction | | 14.7 | 7.4 | 6.7 | 7.1 |
| **10** | **-** | **-** | **-** | **V** | 20.3 | 9.3 | **0** | **0** |
| 14 | V | - | - | V | 27.6 | 29.5 | 7.4 | 20.5 |
| 8 | V | - | - | - | 30.9 | 31.0 | 6.7 | 17.6 |
| 0 | - | - | - | - | 355.4 | 340.7 | 1541 | 1537 |

^a^Models are ranked by ascending AIC for species richness. V/-: Inclusion/omission of this predictor.

^b^Temp: mean annual temperature.

^c^Rain: mean annual rainfall.

^d^Total Butterfly Abundance: sum of all abundance indices (the number of expected observations in adults if the transect was visited once per week) of species occurring in the low-lying Mediterranean ecoregion in 2019.

^e^ΔAIC/BIC is the difference between the model’s AIC/BIC and the minimum AIC/BIC of all nested models. Predictors of models with ΔAIC/BIC < 2 are given in bold.

**Table S1.4.** Generalized linear model selection for Israeli butterfly species abundance and occurrence in the low-lying Mediterranean region using Akaike (AIC) and Bayesian (BIC) criteria^a^

| **Model no.** | **Habitat** | **Soil** | **Temp.^b^** | **Rain^c^** | **Abundance^d^** | | **Occurrence^e^** | |
| --- | --- | --- | --- | --- | --- | --- | --- | --- |
|  |  |  |  |  | **ΔAIC** | **ΔBIC** | **ΔAIC** | **ΔBIC** |
| **15** | **-** | **-** | **Temp., Rain & interaction** | | **0** | 110.5 | 29.6 | 168.2 |
| **11** | **-** | **-** | **V** | **V** | 16.8 | 75.1 | **0** | 48.0 |
| 10 | - | - | - | V | 30.6 | 36.7 | 69.4 | 26.7 |
| 9 | - | - | V | - | 36.4 | 42.6 | 65.4 | 22.7 |
| **0** | **-** | **-** | **-** | **-** | 46.1 | **0** | 133.4 | **0** |
| 13 | - | V | - | V | 82.0 | 505.6 | 555.3 | 1509.8 |
| 5 | - | V | V | V | 89.0 | 564.8 | 497.4 | 1542.6 |
| 7 | - | V | - | - | 111.1 | 482.6 | 547.2 | 1411.1 |
| 6 | - | V | V | - | 115.3 | 538.9 | 508.5 | 1463.0 |
| 3 | V | V | - | V | 128.0 | 864.7 | 754.3 | 2343.4 |
| 1 | V | V | V | V | 131.3 | 920.2 | 622.6 | 2211.8 |
| 2 | V | - | V | V | 140.0 | 563.6 | 308.8 | 1082.1 |
| 12 | V | - | V | - | 145.3 | 516.8 | 357.3 | 1039.9 |
| 14 | V | - | - | V | 160.1 | 531.6 | 364.7 | 1047.3 |
| 4 | V | V | V | - | 162.3 | 899.1 | 748.6 | 2337.8 |
| 8 | V | - | - | - | 178.6 | 497.7 | 429.2 | 1021.1 |

^a^Models are ranked by ascending AIC for species richness. V/-: Inclusion/omission of this predictor.

^b^Temp: mean annual temperature.

^c^Rain: mean annual rainfall.

^d^Abundance: abundance index (number of expected observations in adults if the transect was visited once per week) of species occurring in the low-lying Mediterranean ecoregion in 2019. ΔAIC/BIC is the difference between the model’s AIC/BIC and the minimum AIC/BIC of all nested models. Predictors of models with ΔAIC/BIC < 2 are given in bold.

^e^Occurrence: species list in the first 5 years of monitoring. ΔAIC/BIC is the difference between the model’s AIC/BIC and the minimum AIC/BIC of all nested models. Predictors of models with ΔAIC/BIC < 2 are given in bold.

**Appendix S3: butterfly species’ ecological niches in Israel**

*Papilio machaon* is a widespread and moderately abundant species (Table 2), occurring in all regions of Israel (including the dry and cool Negev Mountains; Benyamini 2010), except for the hyper-arid Wadi Araba. It is probably sensitive to hot temperatures, but not to low rainfall (Fig. 4A). Alternatively, it is also possible that its host plant species (Apiaceae and also *Ruta chalepensis*) are sensitive to hot climates. Within the low-lying Mediterranean ecoregion, it is more abundant in relatively cool habitats with high precipitation (Fig. 4B). It commonly co-occurs with *Lasiomamata maera*, *Pontia daplidice*, *Melanargia titea*, and *Vanessa atalanta* (Fig. 3).

*Archon apollinus* is a relatively widespread species that occurs more often in regions of Israel with high precipitation (Table 1, Fig. 4A). It does not occur in Wadi Araba. It commonly co-occurs with *Satyrium spini*, *Hipparchia fatua*, *Anthocharis cardamines*, and *Gonepteryx cleopatra*, but only rarely with *Euchloe charlonia* or *Deudorix livia* (Fig. 3A).

*Pieris brassicae* is a very widespread and abundant species, but it does not occur in Wadi Araba (Table 1). Within the low-lying Mediterranean region of Israel, it is more abundant warm habitats with high precipitation (e.g., Galilee Coast, Mt. Carmel; Fig. 4B). *P*. *brassicae* commonly co-occurs with *Maniola telmessia*, *Vanessa atalanta*, *Gonepteryx cleopatra*, and *Anthocharis cardamines*, but only rarely with *Pontia glauconome* or *Euchloe charlonia* (Fig. 3).

*Anthocharis cardamines* is restricted to Mediterranean regions of the country, where it is relatively widespread but not abundant (Table 1). It is more abundant in cool, high-precipitation parts of the low-lying Mediterranean ecoregion (e.g., mountain slopes in Upper Galilee). *Colias crocea* and *Gonepteryx cleopatra* have similar climatic niches, yet are more abundant. *C*. *crocea* also occurs in the semi-desert.

*Danaus chrysippus* is an Afro-tropical migrant occurring in transects from all regions of the country except the high Mediterranean (where it is only recorded in sporadic observations and thus is probably very rare; Israel Butterfly Monitoring Scheme 2020), but nowhere in high numbers and not in many transects (Table 1). *D*. *chrysippus* often co-occurs with other warm adapted species such as *Zizeeria karsandra* and *Chilades trochylus* but only rarely with *Hipparchia fatua* (Fig. 3B).

*Vanessa atalanta* is a widespread species, but scarce in terms of abundance (Table 1). It is most abundant in high-precipitation habitats in the low-lying Mediterranean region. *Vanessa cardui* is super-abundant and extremely widespread, not only in Israel (Table 1) but all over the world (e.g., Talavera et al. 2018; Benyamini 2017). Both *Vanessa* species are more abundant in high-precipitation habitats, and *V*. *atalanta* more so (Fig. 4B). *V*. *atalanta* is more associated with other species common in high-precipitation habitats, such as *Pieris brassicae* and *Gonepteryx cleopatra*.

*Hipparchia fatua* is the species that responds most strongly to rainfall (i.e., its numbers change the most with changes in mean annual rainfall; Fig. 4B), even within the low-lying Mediterranean ecoregion. In addition, *H*. *fatua* is less abundant in warm habitats, especially if they are also arid. It is rarely abundant in the same habitats as *Zizeeria karsandra*, *Chilades trochylus*, or *Lycanaea thersamon* (Fig. 3B), despite the association of the latter two species with relatively cool and high-precipitation habitats within the study area (Fig. 6). *Maniola telmessia* is likewise more abundant in high-precipitation habitats within the low-lying Mediterranean ecoregion (Fig. 4B), but is more widespread and abundant than *H*. *fatua* (Table 1). *L*. *maera* is moderately widespread but relatively scarce (Table 1). Unlike *H*. *fatua* and *M*. *telmessia*, *L*. *maera*’s abundance responds more strongly to temperature than to rainfall. Within the study area, it occurs more often in cool, high-precipitation habitats (Fig. 4).

*Deudorix livia* occurs most often in arid yet not very warm habitats (Fig. 4A), and only rarely co-occurs with *Satyrium spini*. It is moderately widespread, although it is rare in the high Mediterranean ecoregion (Table 1).

*Apharitis acamas* is a myrmecophile species whose hosts are *Crematogaster* *jehovae* ants (Benyamini 2010). Although *C*. *jehovae* is distributed from southeastern Europe to the south of the Arabian Peninsula (Sharaf et al. 2019), *A*. *acamas* has a more restricted distribution and is not found in the semi-desert or Wadi Araba (Table 1).

Both *Lycaena* species studied here (*L*. *phlaeas* and *L*. *thersamon*) tend to occur in relatively cool habitats within the study area (Fig. 4A). Of the two congeners, *L*. *thersamon* is considerably more widespread and abundant (Table 1), and its occurrence can be more strongly associated with cooler habitats. *L*. *phlaeas* often co-occurs with *Lasiommata maera*, while *L*. *thersamon* is more often seen in the same habitats as *Chilades trochylus* and *Papilio machaon* (Fig. 3A).

*Tarucus balkanicus* occurs in moderately arid yet not very warm habitats (i.e., not in Araba Wadi), similar to *Deudorix livia* (Fig. 4A). Its occurrence is not strongly correlated with the occurrence of any other species studied, and it is only weakly correlated with *D*. *livia* and *Euchloe charlonia* (Fig. 3A).

*Azanus jesous* occurs in all regions of the country (Table 1), but nevertheless it is more likely to occur along drier transects (Fig. 4A). *A*. *jesous* can be very abundant locally, as is apparent in its high variance in abundance between transects (Table 1). Within the low-lying Mediterranean ecoregion, it is considerably more abundant in warm and relatively arid habitats.

*Chilades trochylus* is a fairly widespread and abundant species, occurring from the semi-desert to the high Mediterranean region (Table 1). It is thus negatively associated with the high temperatures of Araba Wadi (Fig. 4A). It often co-occurs with *Zizeeria karsandra* and *Lycaena thersamon* (Fig. 3B).

*Aricia agestis* is fairly uncommon; it is limited to the Mediterranean ecoregions of the study area (Table 1), and hence to relatively cool habitats (Fig. 4A). It often co-occurs with *Carcharodus alceae* and *Lasiommata maera* (Fig. 3A).

*Polyommatus icarus* occurs in the Mediterranean region, where its abundance varies greatly (Table 1). It is more abundant in relatively cool habitats within the low-lying Mediterranean ecoregion (Fig. 4B). It often co-occurs with *Lasiommata maera* and *Maniola telmessia* (Fig. 3A).

*Zizeeria karsandra* occurs in all of the ecoregions studied, but not in many transects; its abundance varies greatly (Table 1). It tends to occur more along relatively arid transects (Fig. 4A). It is often relatively abundant in the same habitats as *Lycaena thersamon* and *Chilades trochylus*.

**References**

Benyamini, D. 2010. A Field Guide to the Butterflies of Israel, Including Mt. Hermon, Sinai and Jordan (Revised edition). – Keter Publishing House, Jerusalem, Israel.

Benyamini, D. 2017. A swarm of millions of Vanessa cardui (Linnaeus, 1758) in winter-spring 2015-2016 in the south-east Mediterranean - The missing link (Lepidoptera, Nymphalidae). – Atalanta **48** (1-4): 103-128.

Israel Butterfly Monitoring Scheme (BMS-IL). 2020. Sightings data (online database). Accessed May 31^st^, 2020. URL: <http://www.gluecad-bio.com/hompage.asp?lng=eng>

Sharaf, M. R. et al. 2019. Review of the Arabian Crematogaster Lund (Hymenoptera, Formicidae), synoptic list, distribution, and description of two new species from Oman and Saudi Arabia. – ZooKeys 898: 27-81. <http://doi.org/10.3897/zookeys.898.37531>

Talavera, G. et al. 2018. Round-trip across the Sahara: Afrotropical Painted Lady butterflies recolonize the Mediterranean in early spring. – Biol. Lett. **14**: 20180274. http://dx.doi.org/10.1098/rsbl.2018.0274
